# Low-Crosslinked Hyaluronic Acid Injections in the Superficial Fat Layer for Facial Rejuvenation in Chinese Patients: A Retrospective Clinical Study

**DOI:** 10.7759/cureus.79607

**Published:** 2025-02-25

**Authors:** Xiaohui Wu, Bo Wang, Yong Liao, Xiaojuan Li, Jingjing Chen, Liangsen Zhao

**Affiliations:** 1 Medicine, Bloomage Biotechnology Corporation Limited, Beijing, CHN; 2 Plastic Surgery, Beijing Berrina Medical Aesthetic Clinic, Beijing, CHN; 3 Dermatology, Beijing Berrina Medical Aesthetic Clinic, Beijing, CHN

**Keywords:** facial rejuvenation, retinacula cutis, skin rejuvenation, superficial fat layer, hyaluronic acid

## Abstract

Objective

To evaluate the clinical efficacy of low-crosslinked hyaluronic acid (HA) injections in the superficial fat layer for facial rejuvenation in Chinese patients.

Methods

A total of 30 patients were treated between July 2023 and October 2024, with three sessions of low-crosslinked HA injections spaced one month apart. The injections were administered using the fanning technique into the superficial fat layer. Patients were followed up at one month and three months post-treatment. Skin improvements were assessed using smart skin analysis equipment, while overall aesthetic improvement was evaluated using the Global Aesthetic Improvement Scale (GAIS). Patient pain levels, satisfaction, and adverse reactions were also recorded.

Results

Significant improvements in facial wrinkles, pore size, and pigmentation were observed at one and three months post-treatment, as measured by skin analysis equipment, compared to baseline. GAIS scores confirmed facial rejuvenation in all patients. Patient satisfaction was 100% at one month and 93.3% at three months. Mild discomfort was reported by 90% of patients, with an average pain score of 3.40 ± 1.55, resolving within 24 hours. Two patients experienced mild bruising, which resolved within a week. No other adverse reactions were noted.

Conclusion

Low-crosslinked HA injection in the superficial fat layer is an effective and safe method for facial rejuvenation, offering high patient satisfaction. No serious adverse events were reported during the follow-up period.

## Introduction

Skin aging is one of the most apparent and characteristic changes in the aging process, manifested by clinical signs such as dryness, roughness, fine lines, wrinkles, facial volume loss, decreased elasticity, reduced firmness, skin sagging, enlarged pores, and loss of radiance [1,2]. Conventional facial skin rejuvenation injectables primarily target the dermal layer, aiming to improve skin texture by modifying the activity of dermal fibroblasts and remodeling the extracellular matrix (ECM) [3,4]. Recent studies on facial skin aging have shown that the subcutaneous fat tissue and the retinacula cutis (RC) beneath the dermis also play significant roles in the aging process, contributing to the loss of facial contour smoothness, wrinkles, sagging, and laxity [5-7].

Injection of low-crosslinked hyaluronic acid (HA) into the dermis has proven to be highly effective in skin rejuvenation, directly replenishing HA, enhancing skin hydration, increasing mechanical tension on fibroblasts, and promoting collagen synthesis. These lead to improvements in skin texture, dryness, roughness, and luminosity, restoring a youthful appearance [3,4]. However, there is limited clinical research on the injection of low-crosslinked HA into the facial superficial fat layer. This study aims to explore the safety and efficacy of low-crosslinked HA injections into the superficial fat layer in facial rejuvenation treatments.

## Materials and methods

Selection of study patients

This study collected data from 30 female patients aged 28-67 years (mean age 44.73 ± 11.66 years), who presented with signs of facial skin aging at the Beijing Berrina Medical Aesthetic Clinic between October 2023 and May 2024. The study cohort consisted of patients with Fitzpatrick skin types II to IV [8].

The inclusion criteria for the study are as follows: patients who are seeking facial rejuvenation treatment; patients with moderate (type II), advanced (type III), or severe (type IV) skin photoaging as classified by the Glogau Photoaging Scale [9]; patients who have provided signed informed consent; and patients who demonstrate good compliance and are capable of completing a three-month post-treatment follow-up.

The exclusion criteria for this study encompass the following: individuals who are pregnant, planning to become pregnant, or in the lactation period; patients with active skin infections; individuals suffering from autoimmune diseases; patients with severe systemic conditions; those with clotting disorders; patients with a history of abnormal scarring; individuals with known allergic conditions; patients who are currently undergoing other facial rejuvenation treatments; and individuals with mental health issues or those holding unrealistic expectations regarding the treatment outcomes.

Materials

The low-crosslinked HA used in this study was an injectable modified sodium hyaluronate gel (Aqua type, 2 mL, Shandong Food and Drug Administration Equipment Production Permit No. 20120123, Bloomage Biotechnology Corporation Limited, Beijing, China). The topical anesthetic used was a compound lidocaine cream (2.5% lidocaine and 2.5% prilocaine, Beijing Ziguang Pharmaceutical Co., Ltd., Beijing, China).

Treatment protocol

First, the patient's face was thoroughly cleaned, and a digital photograph was taken for documentation. Digital photographs were taken from the front, at 45°, and at 90° angles, ensuring that consistent lighting and background conditions were maintained for accurate documentation. Next, the physician made a pre-treatment assessment of the patient's skin aging condition. The patient’s facial skin aging was graded according to the Glogau Photoaging Scale. Based on the aging grade, the initial injection dose was determined as follows: 4 mL for type II (moderate), 8-12 mL for type III (advanced), and 12 mL for type IV (severe). For the second and third sessions, the injection dose was adjusted according to the patient’s specific facial condition and response to previous treatments.

**Figure 1:**
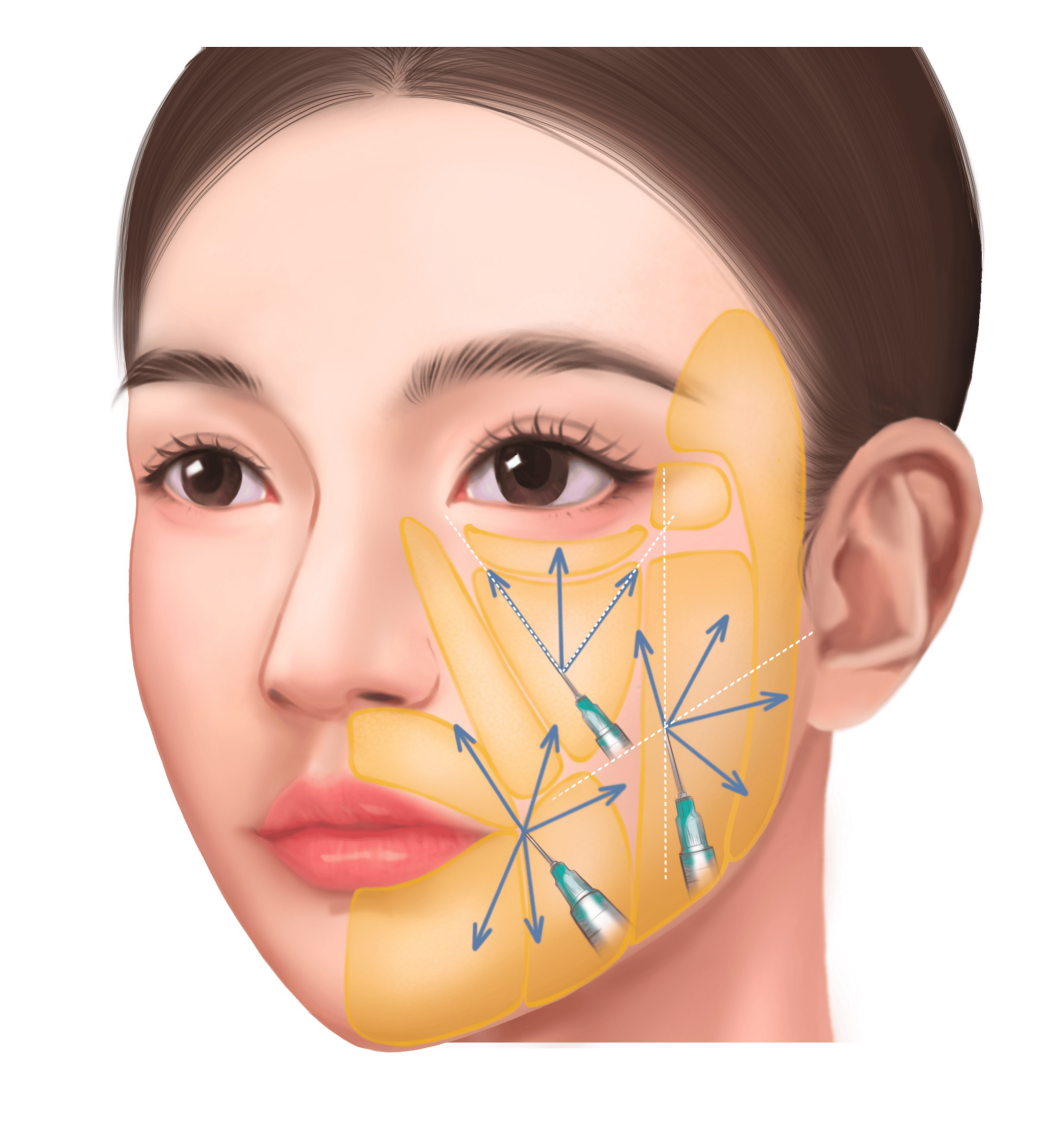
Schematic figure of low-crosslinked hyaluronic acid (HA) application in the mid-face and lower face. Blue vectors: fanning injection technique in the superficial fat layer according to the authors’ recommendation. Image credit: The figure was drawn by the team, referring to a picture in the literature [10].

Following the application and removal of local anesthesia, which was left for 30 minutes, the face was disinfected in preparation for injections. Using a 25G, 50 mm cannula, low-crosslinked HA was injected into three specific areas: the suborbital region, where the cannula was inserted 1-2 cm below the infraorbital rim along the vertical line from the outer canthus, with 0.1 mL of HA injected per line in a fan-shaped linear pattern at the superficial fat layer; the lateral cheek, where the cannula was inserted at the intersection of the vertical line from the outer canthus and the line connecting the tragus to the corner of the mouth, again with 0.1 mL per line in a fan-shaped distribution; and the perioral area, where the cannula was inserted at least 2 cm lateral to the oral commissure, with the same technique applied. After each injection, a light massage was performed to ensure even distribution of the product. A treatment diagram illustrating these points is provided in Figure 1.

The treatment consisted of three sessions, each spaced one month apart. The injection dose for each session was determined based on the patient's skin condition and the physician’s pre-treatment assessment. The time, site, and dose of each injection were recorded. Follow-up evaluations were conducted at one month (M1) and three months (M3) after the final treatment.

Observation parameters

The physician evaluated the treatment's efficacy using the Global Aesthetic Improvement Scale (GAIS) [11], with categories ranging from "Very Much Improved," indicating an optimal cosmetic result with exceptional and natural-looking improvement, to "Much Improved," reflecting a marked improvement in appearance from the initial condition, but not completely optimal for this subject. "Improved" denotes obvious improvement in appearance from the initial condition, but re-treatment is indicated, while "No Change" signifies that the appearance is essentially the same as the original condition. Conversely, "Worse" indicates that the appearance is worse than the original condition.

The ALURA LEGEND Smart Skin Analyzer (Allura, Houston, TX, USA) was used to capture facial photographs from the front, left 45°, and right 45° angles after cleaning the patient’s face and allowing them a 10-minute rest. Skin status was analyzed at both pre-treatment and follow-up visits, with a lower score indicating a better facial skin condition.

Patient satisfaction was classified into four levels: very satisfied, satisfied, somewhat satisfied, and dissatisfied. The satisfaction rate was calculated as follows: Satisfaction rate = (very satisfied + satisfied cases)/total cases × 100%.

Patients self-reported their pain level during the procedure using the Visual Analog Scale (VAS) [12]. The VAS is a validated and widely used tool for measuring subjective pain intensity. It consists of a horizontal or vertical line, typically 10 cm in length, with two endpoints labeled "0" and "10." In this study, patients were asked to mark their pain level on the scale, where 0 represented "no pain" and 10 represented "intolerable pain." The VAS score was recorded as the distance in centimeters from the "0" endpoint to the patient's mark, providing a continuous measure of pain intensity. In addition, adverse reactions, including erythema, bruising, and edema, were observed and recorded during the treatment.

Statistical analysis was performed using IBM SPSS Statistics for Windows, Version 25 (Released 2017; IBM Corp., Armonk, NY, USA). Continuous data, including wrinkle depth, superficial pigmentation scores, and pore size, were expressed as means ± standard deviations (x ± s) and analyzed using paired t-tests to compare the results before treatment, at one month post-treatment, and at three months post-treatment. Categorical data, such as patient satisfaction rates, were expressed as percentages (%) and presented descriptively. A p-value of <0.05 was considered statistically significant.

## Results

Efficacy evaluation

**Figure 2:**
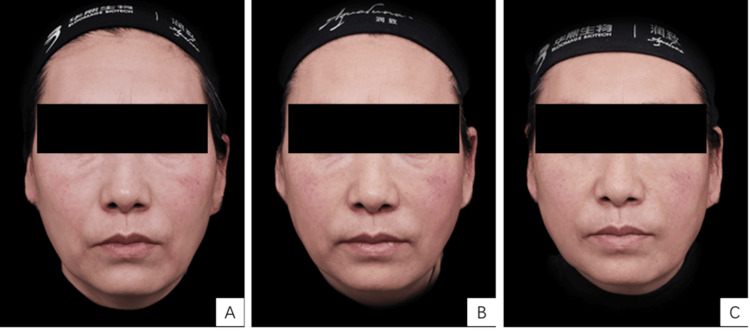
Photos of the patient before and after treatment. (A) Pre-treatment; (B) One month post-treatment; (C) Three months post-treatment A 60-year-old female with advanced photoaging, classified as type III, on the Glogau Photoaging Scale, underwent three sessions with low-crosslinked hyaluronic acid injections, receiving 12 mL, 8 mL, and 8 mL, respectively. The patient consented and a written and signed consent statement was provided to the journal.

The GAIS showed varying degrees of improvement in facial skin condition across all participants when compared to baseline (M0). One month after the final treatment (M1), 25 patients (83.3%) reported a "much improved" outcome, while five patients (16.7%) experienced an "improved" condition. At three months post-treatment (M3), seven patients (23.3%) demonstrated a "very much improved" result, 20 patients (66.7%) were categorized as "much improved," and three patients (10%) showed an "improved" condition. The detailed results are summarized in Table 1, and representative patient photos before and after treatment are provided in Figure 2.

**Table 1:** Global Aesthetic Improvement Scale (GAIS) assessment. M1: One month after the final treatment; M3: Three months post-treatment Very Much Improved, indicated an optimal cosmetic result with exceptional and natural-looking improvement; Much Improved, reflected a marked improvement in appearance from the initial condition, but not completely optimal for this subject; Improved, denotes obvious improvement in appearance from initial condition, but a re-treatment is indicated.

	Improved	Much improved	Very much improved
M3	10%	66.7%	23.3%
M1	16.7%	83.30%	-

ALURA LEGEND Smart Skin analysis

**Figure 3:**
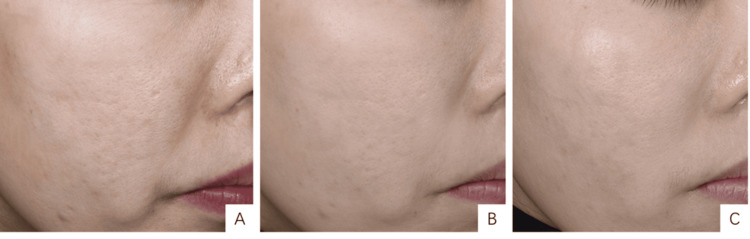
ALURA LEGEND Smart Skin Assessment - wrinkle severity percentage. (A) Pre-treatment, with a wrinkle severity percentage of 13.8%; (B) One month post-treatment, with a severity percentage of 12.8%; (C) Three months after treatment, with a severity percentage of 10.99%. A 33-year-old female patient, exhibiting moderate signs of aging, classified as type II on the Glogau Photoaging scale, underwent three treatment sessions, each receiving 4 mL of low-crosslinked hyaluronic acid (HA).

**Figure 4:**
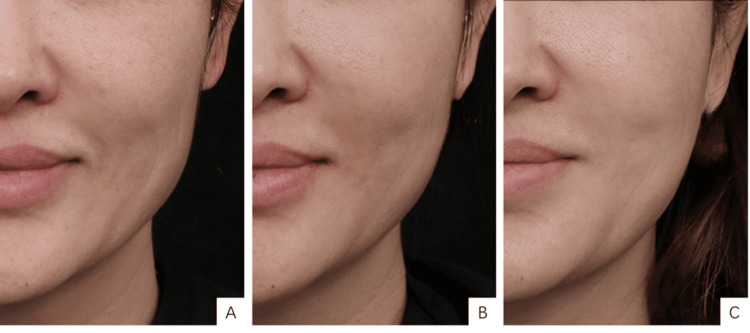
ALURA LEGEND Smart Skin Analysis - wrinkle severity percentage. (A) Pre-treatment percentage of severe wrinkles, at 15.51%; (B) One month post-treatment, showing the percentage reduced to 9.02%; (C) Three months post-treatment, showing a further reduction to 7.66%. A 53-year-old female patient, presenting with advanced signs of aging and classified as type III on the Glogau Photoaging Scale, received three treatment sessions with low-crosslinked hyaluronic acid (HA) injections, totaling 8 mL, 4 mL, and 4 mL, respectively.

**Figure 5:**
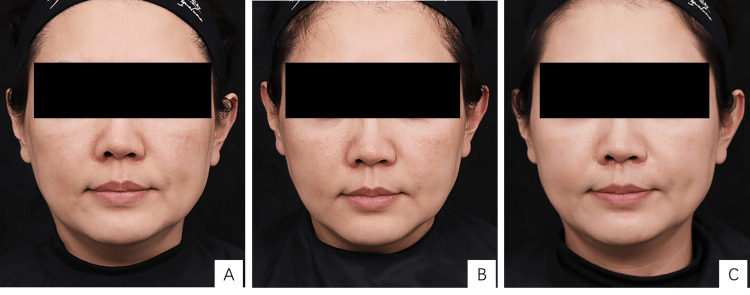
ALURA LEGEND Smart Skin Analysis - superficial skin pigmentation. (A) Pre-treatment percentage of superficial skin pigmentation at 17.16%; (B) One month post-treatment, showing that the percentage was reduced to 15.24%; (C) Three months post-treatment, showing a further reduction to 14.93%. A 42-year-old female patient, presenting with advanced signs of aging and classified as type III on the Glogau Photoaging Scale, received three treatment sessions with low-crosslinked hyaluronic acid (HA) injections. The total volumes of HA injected were 10 mL, 8 mL, and 4 mL, respectively, across the three sessions. The patient consented and a written and signed consent statement was provided to the journal.

The analysis conducted by the ALURA LEGEND Smart Skin Detection system on patients' wrinkles, pores, and surface pigmentation, before and after treatment, revealed significant improvements in all parameters compared to pre-treatment values (Figures 3-5). These differences were statistically significant, as shown in Table 2.

**Table 2:** ALURA LEGEND Smart Skin Analysis data *Compared to pre-treatment (M0), p < 0.05; t compared to pre-treatment (M0).

	Pre-treatment (M0)	1 month post-treatment (M1)	t	p-value	3 months post-treatment (M3)	t	p-value
Wrinkles (%)	19.56 ± 6.59	15.18 ± 5.96	4.104	0.003*	12.07 ± 5.68	4.968	0.001*
Pores (%)	30.01 ± 9.28	26.88 ± 7.56	3.565	0.006*	23.18 ± 7.23	5.987	0*
Superficial skin pigmentation (%)	16.45 ± 3.48	15.29 ± 3.00	3.549	0.006*	13.90 ± 2.02	3.688	0.005*

Patient satisfaction rate

One month after the treatment course, among the 30 patients, 12 were very satisfied, 15 were satisfied, and three were somewhat satisfied, resulting in an overall satisfaction rate of 90%. Three months after the treatment, during follow-up, 15 patients were very satisfied, 13 were satisfied, and two were somewhat satisfied, yielding an overall satisfaction rate of 93.3%.

Pain scores and adverse reactions

During the injection process, 27 patients (90%) reported mild pain, with a VAS score ranging from 2 to 3; three patients (10%) experienced moderate pain, with a VAS score ranging from 4 to 6. The mean VAS score for all subjects during the procedure was 3.40 ± 1.55. All pain subsided within 24 hours. Two patients developed bruising near the injection site after the treatment, which resolved spontaneously within one week. No other adverse reactions were observed during follow-up.

## Discussion

With aging, skin tissue undergoes degenerative changes at both the cellular and ECM levels, leading to various clinical manifestations. The key aspects of skin aging typically include the formation of wrinkles, characterized by the gradual appearance of fine lines and deeper folds, which are among the most prominent signs. Additionally, sagging and loss of elasticity occur as the skin begins to droop and lose its firmness. Changes in skin texture are also evident, with the skin becoming rougher and pores often becoming more enlarged. Pigmentation changes, such as the development of age spots, lentigines, freckles, and uneven skin tone, further contribute to the aging process [13-15].

HA, a bioactive substance, plays multiple physiological roles. It functions as a signaling molecule involved in various signaling pathways, with effects such as inhibiting inflammation, providing antioxidant protection, promoting wound healing, and enhancing angiogenesis. Additionally, HA serves as a major component of the ECM, contributing to the maintenance of hydration, viscoelasticity, and structural support. These characteristics make HA a key ingredient in skin rejuvenation injection treatments [16-18]. Traditionally, HA products have been injected into the dermis to improve skin texture, hydration, and fine lines. However, clinical results involving deeper skin issues, such as contour irregularities, skin laxity, and sagging, have been less effective [19-21]. Recent clinical studies have confirmed that HA injections in a subcutaneous layer can restore facial superficial volume and improve the structure of superficial connective tissue, including the dermis and RC [22-24]. However, related clinical data from the Chinese population remain limited.

This retrospective study evaluated the efficacy and safety of low-crosslinked HA injections in the superficial fat layer for facial rejuvenation in Chinese patients. The outcomes demonstrated substantial enhancements in facial skin texture and fullness at one-month and three-month follow-ups compared to pre-treatment. Improvements in skin texture comprised increased hydration, glossiness, smoothness, and a reduction in fine lines and wrinkles. Furthermore, facial fullness, elasticity, and firmness also saw significant improvements. Results from the ALURA LEGEND Smart Skin Analyzer indicated improvements in skin wrinkles, pores, and pigmentation, with statistically significant differences compared to baseline. These effects can be attributed to the characteristics of the low-crosslinked HA product used in this study, which contains small particles (200 µm), a low crosslinking degree (0.6% degree of crosslinking), and specific viscoelastic properties. Compared to non-crosslinked HA, this formulation offers better volume restoration, longer-lasting effects, and mild stimulation of fibroblasts, facilitating the remodeling of collagen and elastin in the skin ECM.

Research has shown that there are no significant anatomical differences between the subcutaneous connective tissue and the dermis. Immunofluorescence studies have revealed similar collagen and elastin content and proportions in both layers, suggesting their homology [25-27]. RC, located between the superficial fascia and dermis, are connective tissue fibers that provide structural support. With aging, the density of these ligaments decreases, leading to reduced subcutaneous tissue density, ECM atrophy, and loss of structural support, resulting in deeper wrinkles, decreased skin elasticity, and more pronounced facial sagging [28,29]. Low-crosslinked HA injected into the superficial subcutaneous layer may help improve the structure and function of RC by promoting collagen synthesis, thus enhancing facial fullness and reducing skin laxity and sagging.

Although preliminary results indicate promising safety and efficacy, there are limitations to the current study. For instance, all patients experienced some degree of pain during treatment; however, most reported only mild discomfort, which was tolerable and resolved within 24 hours post-treatment. No significant adverse reactions, such as embolism or necrosis, were observed, confirming the safety of this treatment. This finding is in line with previous studies on the safety of HA injections, which generally report a low incidence of severe adverse events. However, it is important to note that our study did not include objective assessments of pain intensity or duration, which could be addressed in future research.

Patient satisfaction was high at both the one-month and three-month follow-ups, with a satisfaction rate of 93.3% at three months, which was higher than the 90% observed at one month. This suggests that low-crosslinked HA injections are both effective and long-lasting. In an expert panel consensus, researchers injected low-crosslinked HA into the dermis or subcutaneous tissue and also achieved effective and lasting improvements in facial skin texture and skin quality [30].

Further research is needed to evaluate the histological changes in superficial fat and connective tissue following injection treatments. Additionally, objective assessments of skin elasticity, collagen content, and histological analysis were not conducted in this study. This is a significant limitation, as these parameters are crucial for understanding the mechanisms of action and long-term benefits of low-crosslinked HA injections. Previous studies have shown that HA can promote collagen synthesis and improve skin elasticity; however, the specific effects of low-crosslinked HA remain unclear. Future research should incorporate these assessments to provide a more comprehensive evaluation of treatment efficacy.

There are several limitations to this study. First, the relatively small sample size may introduce statistical bias and limit the generalizability of the findings. Second, objective assessments of skin elasticity, collagen content, and histological analysis were not conducted. Finally, the study was conducted at a single medical institution, which may limit the diversity of the patient population. Future research, involving a broader pool from other medical institutions, will aim to expand upon these findings and provide a more reliable evaluation of the clinical potential of low-crosslinked HA by subcutaneous injection.

## Conclusions

In conclusion, this study demonstrates that subcutaneous injection of low-crosslinked HA can effectively address the clinical manifestations of facial aging. The treatment could improve skin texture, facial wrinkles, pores, and pigmentation. Additionally, the high tolerability and patient satisfaction rates observed in this study further highlight the therapeutic potential of low-crosslinked HA injections in facial rejuvenation. These findings collectively suggest that this approach may serve as a valuable option in the clinical management of facial aging.
